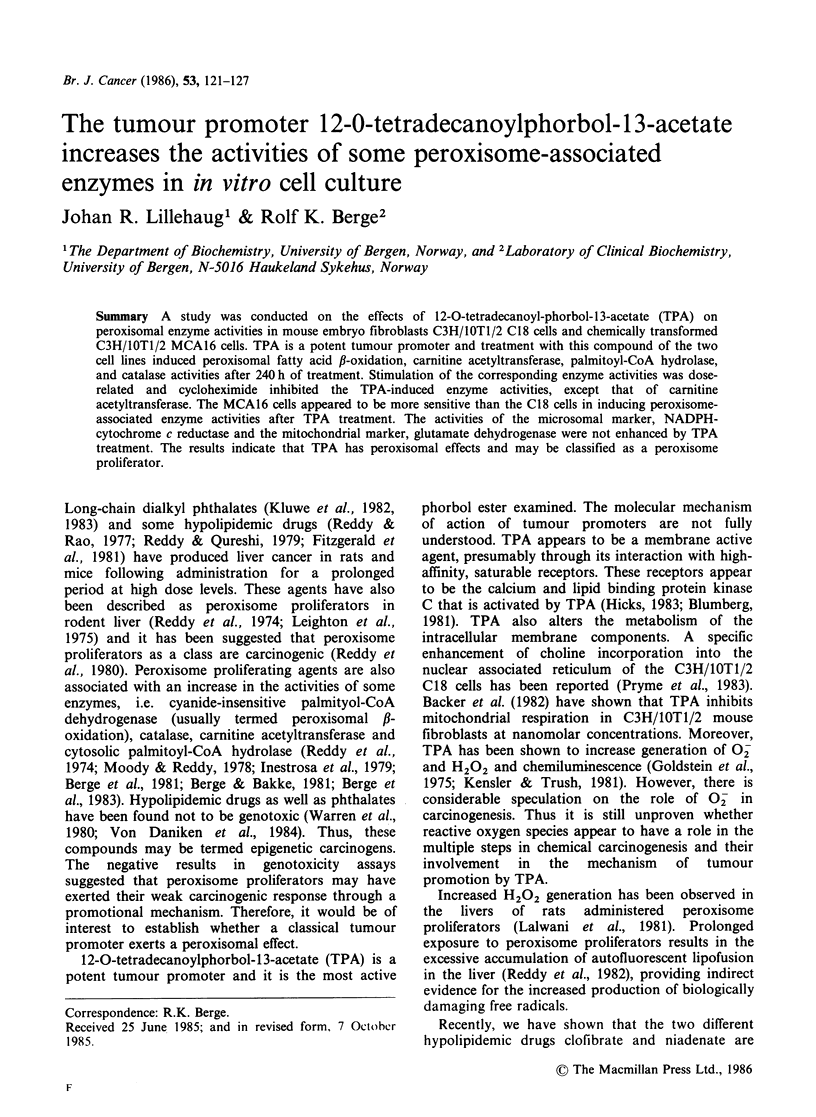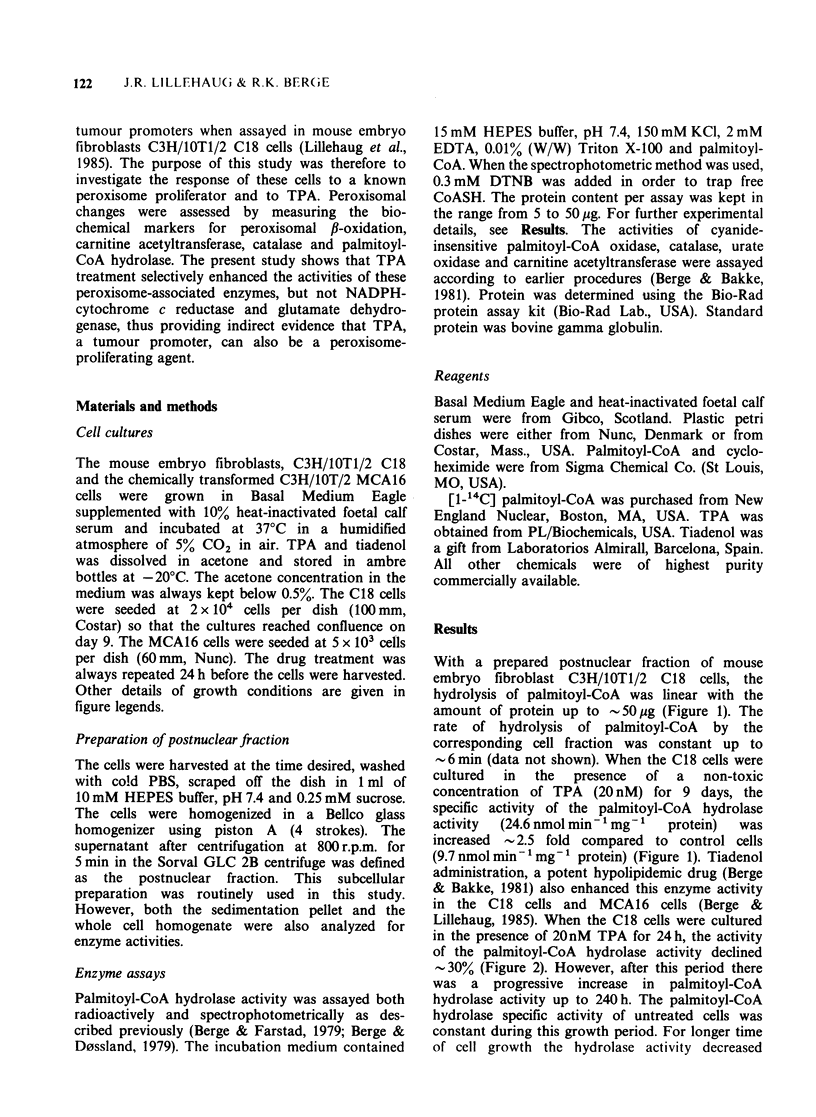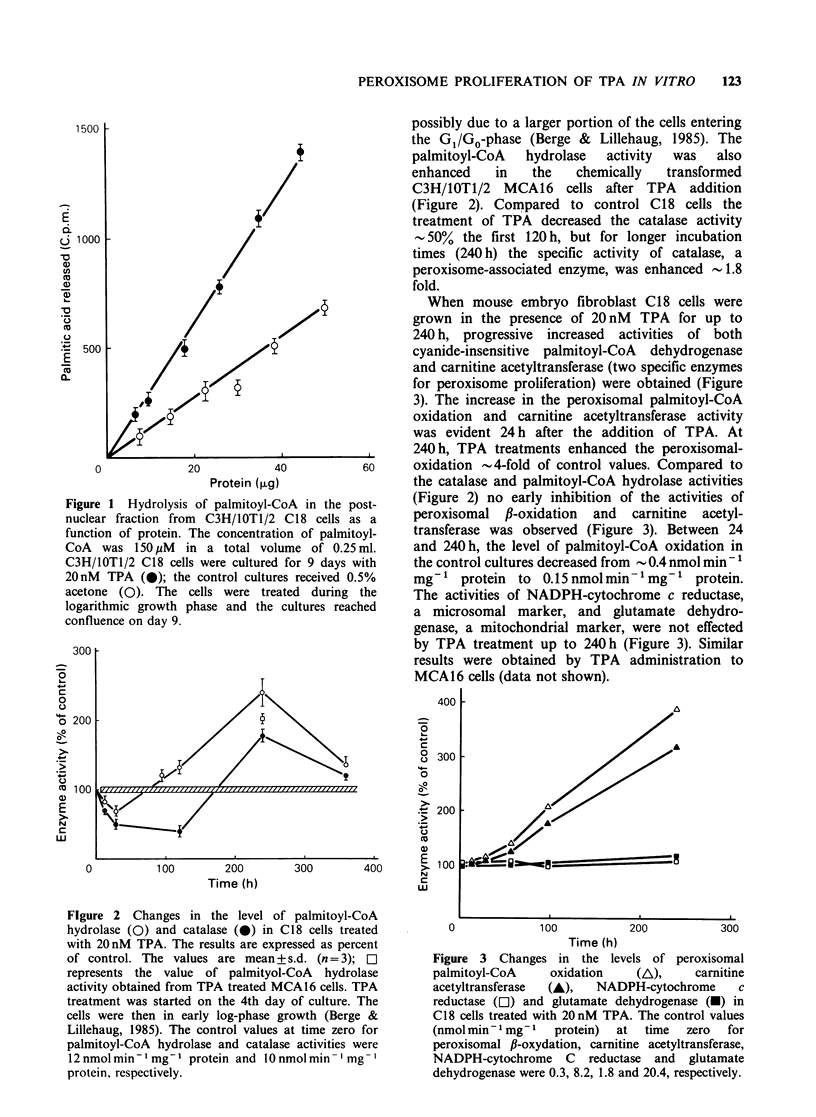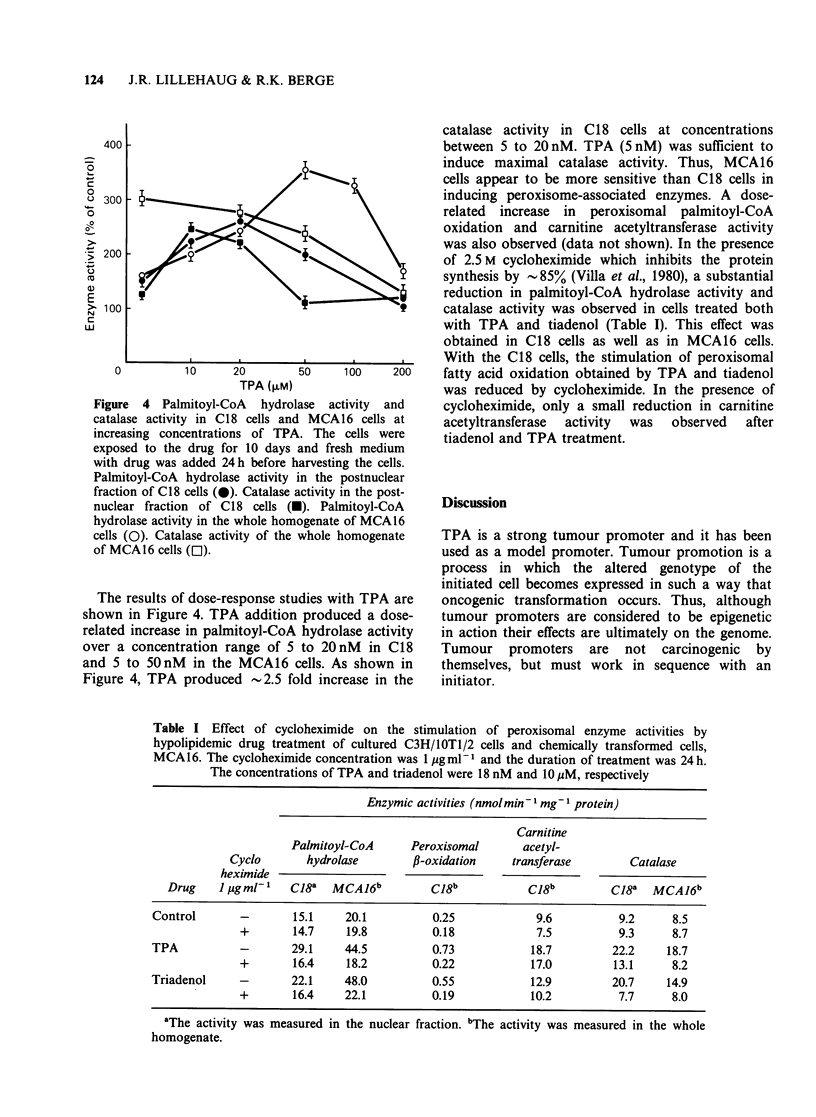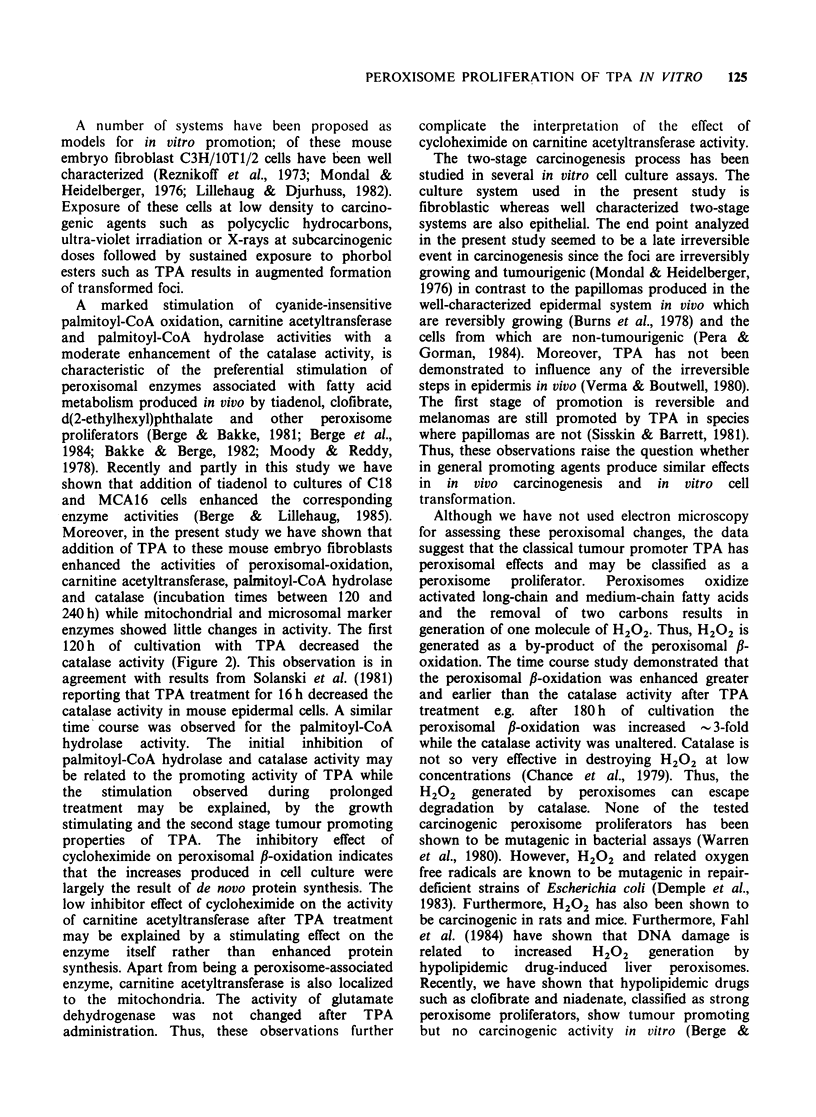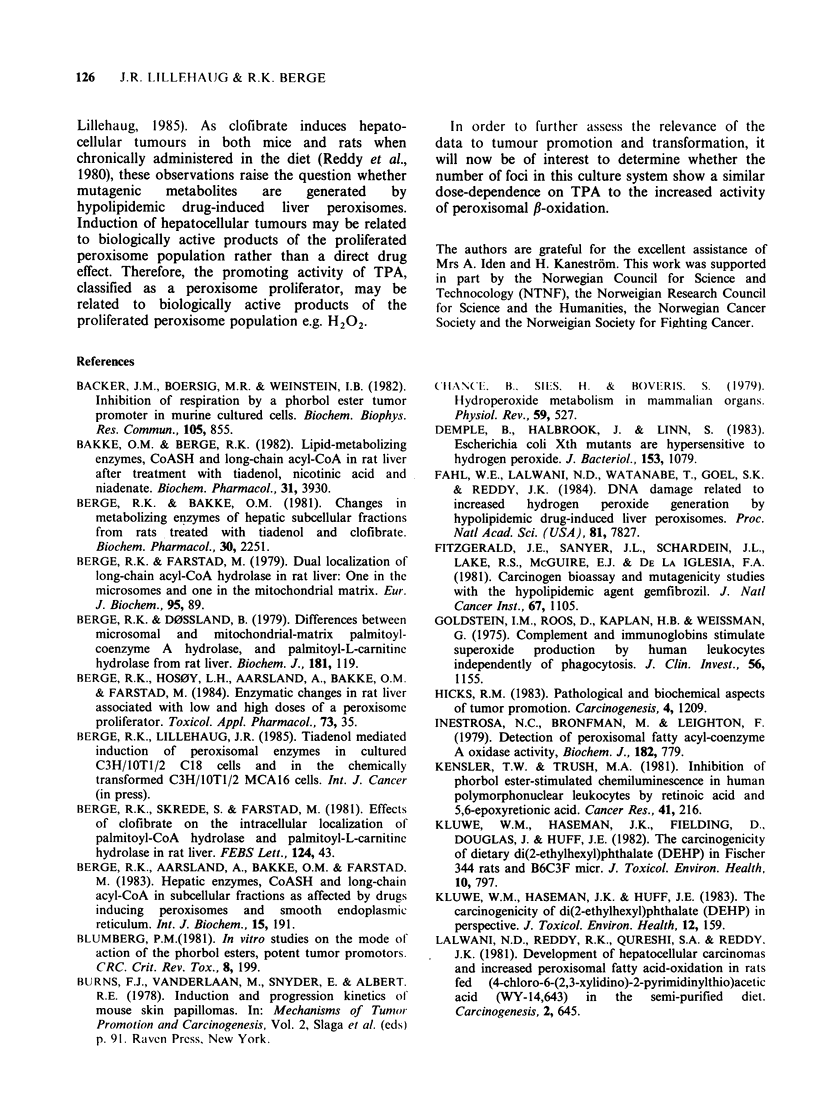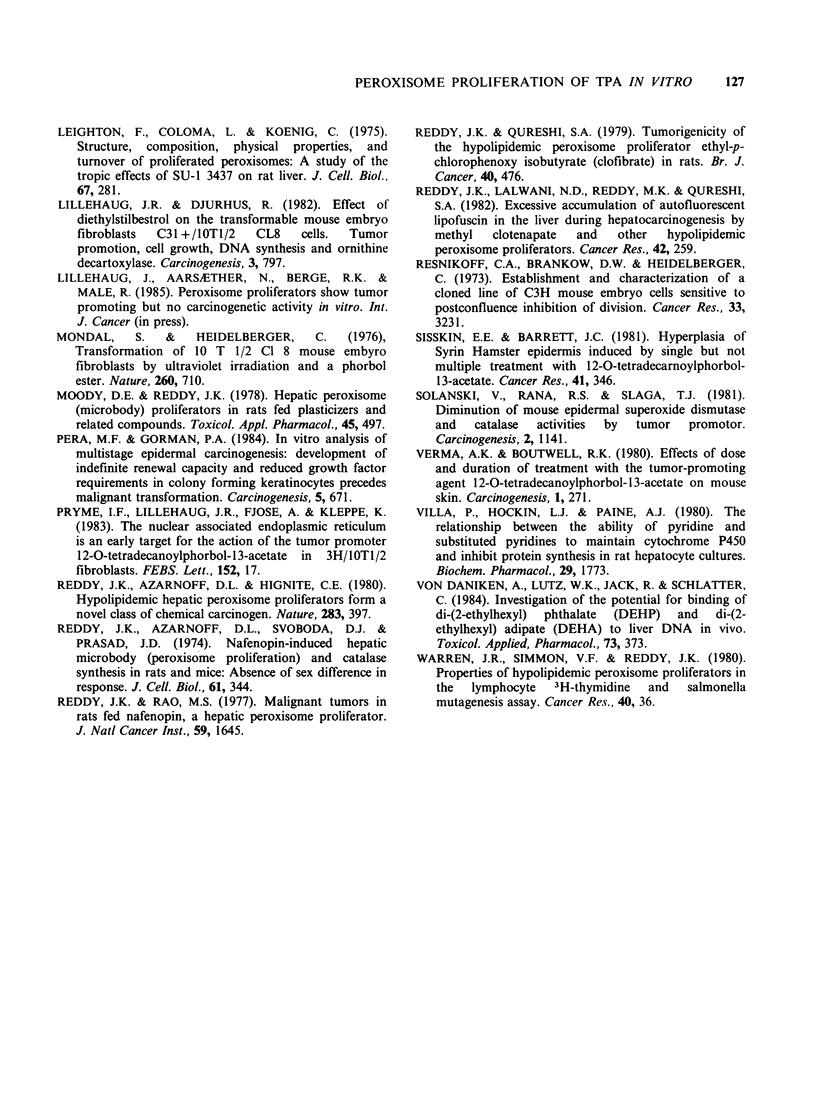# The tumour promoter 12-O-tetradecanoylphorbol-13-acetate increases the activities of some peroxisome-associated enzymes in in vitro cell culture.

**DOI:** 10.1038/bjc.1986.17

**Published:** 1986-01

**Authors:** J. R. Lillehaug, R. K. Berge

## Abstract

A study was conducted on the effects of 12-O-tetradecanoyl-phorbol-13-acetate (TPA) on peroxisomal enzyme activities in mouse embryo fibroblasts C3H/10T1/2 C18 cells and chemically transformed C3H/10T1/2 MCA16 cells. TPA is a potent tumour promoter and treatment with this compound of the two cell lines induced peroxisomal fatty acid beta-oxidation, carnitine acetyltransferase, palmitoyl-CoA hydrolase, and catalase activities after 240 h of treatment. Stimulation of the corresponding enzyme activities was dose-related and cycloheximide inhibited the TPA-induced enzyme activities, except that of carnitine acetyltransferase. The MCA16 cells appeared to be more sensitive than the C18 cells in inducing peroxisome-associated enzyme activities after TPA treatment. The activities of the microsomal marker, NADPH-cytochrome c reductase and the mitochondrial marker, glutamate dehydrogenase were not enhanced by TPA treatment. The results indicate that TPA has peroxisomal effects and may be classified as a peroxisome proliferator.


Br. J. Cancer (1986), 53, 121–127

### The tumour promoter 12-0-tetradecanoylphorbol-13-acetate increases the activities of some peroxisome-associated enzymes in *in vitro* cell culture

Johan R. Lillehaug[1] & Rolf K. Berge[2]

[1]*The Department of Biochemistry, University of Bergen, Norway, and* [2]*Laboratory of Clinical Biochemistry, University of Bergen, N-5016 Haukeland Sykehus, Norway*

**Summary** A study was conducted on the effects of 12-O-tetradecanoyl-phorbol-13-acetate (TPA) on peroxisomal enzyme activities in mouse embryo fibroblasts C3H/10T1/2 C18 cells and chemically transformed C3H/10T1/2 MCA16 cells. TPA is a potent tumour promoter and treatment with this compound of the two cell lines induced peroxisomal fatty acid $\beta$-oxidation, carnitine acetyltransferase, palmitoyl-CoA hydrolase, and catalase activities after 240 h of treatment. Stimulation of the corresponding enzyme activities was dose-related and cycloheximide inhibited the TPA-induced enzyme activities, except that of carnitine acetyltransferase. The MCA16 cells appeared to be more sensitive than the C18 cells in inducing peroxisome-associated enzyme activities after TPA treatment. The activities of the microsomal marker, NADPH-cytochrome *c* reductase and the mitochondrial marker, glutamate dehydrogenase were not enhanced by TPA treatment. The results indicate that TPA has peroxisomal effects and may be classified as a peroxisome proliferator.

Long-chain dialkyl phthalates (Kluwe *et al.*, 1982, 1983) and some hypolipidemic drugs (Reddy & Rao, 1977; Reddy & Qureshi, 1979; Fitzgerald *et al.*, 1981) have produced liver cancer in rats and mice following administration for a prolonged period at high dose levels. These agents have also been described as peroxisome proliferators in rodent liver (Reddy *et al.*, 1974; Leighton *et al.*, 1975) and it has been suggested that peroxisome proliferators as a class are carcinogenic (Reddy *et al.*, 1980). Peroxisome proliferating agents are also associated with an increase in the activities of some enzymes, i.e. cyanide-insensitive palmityol-CoA dehydrogenase (usually termed peroxisomal $\beta$-oxidation), catalase, carnitine acetyltransferase and cytosolic palmitoyl-CoA hydrolase (Reddy *et al.*, 1974; Moody & Reddy, 1978; Inestrosa *et al.*, 1979; Berge *et al.*, 1981; Berge & Bakke, 1981; Berge *et al.*, 1983). Hypolipidemic drugs as well as phthalates have been found not to be genotoxic (Warren *et al.*, 1980; Von Daniken *et al.*, 1984). Thus, these compounds may be termed epigenetic carcinogens. The negative results in genotoxicity assays suggested that peroxisome proliferators may have exerted their weak carcinogenic response through a promotional mechanism. Therefore, it would be of interest to establish whether a classical tumour promoter exerts a peroxisomal effect.

12-O-tetradecanoylphorbol-13-acetate (TPA) is a potent tumour promoter and it is the most active phorbol ester examined. The molecular mechanism of action of tumour promoters are not fully understood. TPA appears to be a membrane active agent, presumably through its interaction with high-affinity, saturable receptors. These receptors appear to be the calcium and lipid binding protein kinase C that is activated by TPA (Hicks, 1983; Blumberg, 1981). TPA also alters the metabolism of the intracellular membrane components. A specific enhancement of choline incorporation into the nuclear associated reticulum of the C3H/10T1/2 C18 cells has been reported (Pryme *et al.*, 1983). Backer *et al.* (1982) have shown that TPA inhibits mitochondrial respiration in C3H/10T1/2 mouse fibroblasts at nanomolar concentrations. Moreover, TPA has been shown to increase generation of $O_2^-$ and $H_2O_2$ and chemiluminescence (Goldstein *et al.*, 1975; Kensler & Trush, 1981). However, there is considerable speculation on the role of $O_2^-$ in carcinogenesis. Thus it is still unproven whether reactive oxygen species appear to have a role in the multiple steps in chemical carcinogenesis and their involvement in the mechanism of tumour promotion by TPA.

Increased $H_2O_2$ generation has been observed in the livers of rats administered peroxisome proliferators (Lalwani *et al.*, 1981). Prolonged exposure to peroxisome proliferators results in the excessive accumulation of autofluorescent lipofusion in the liver (Reddy *et al.*, 1982), providing indirect evidence for the increased production of biologically damaging free radicals.

Recently, we have shown that the two different hypolipidemic drugs clofibrate and niadenate are

Correspondence: R.K. Berge.
Received 25 June 1985; and in revised form, 7 October 1985.

© The Macmillan Press Ltd., 1986

tumour promoters when assayed in mouse embryo fibroblasts C3H/10T1/2 C18 cells (Lillehaug *et al.*, 1985). The purpose of this study was therefore to investigate the response of these cells to a known peroxisome proliferator and to TPA. Peroxisomal changes were assessed by measuring the biochemical markers for peroxisomal $\beta$-oxidation, carnitine acetyltransferase, catalase and palmitoyl-CoA hydrolase. The present study shows that TPA treatment selectively enhanced the activities of these peroxisome-associated enzymes, but not NADPH-cytochrome *c* reductase and glutamate dehydrogenase, thus providing indirect evidence that TPA, a tumour promoter, can also be a peroxisome-proliferating agent.

### Materials and methods

### *Cell cultures*

The mouse embryo fibroblasts, C3H/10T1/2 C18 and the chemically transformed C3H/10T/2 MCA16 cells were grown in Basal Medium Eagle supplemented with 10% heat-inactivated foetal calf serum and incubated at 37°C in a humidified atmosphere of 5% $CO_2$ in air. TPA and tiadenol was dissolved in acetone and stored in ambre bottles at −20°C. The acetone concentration in the medium was always kept below 0.5%. The C18 cells were seeded at $2 \times 10^4$ cells per dish (100 mm, Costar) so that the cultures reached confluence on day 9. The MCA16 cells were seeded at $5 \times 10^3$ cells per dish (60 mm, Nunc). The drug treatment was always repeated 24 h before the cells were harvested. Other details of growth conditions are given in figure legends.

### *Preparation of postnuclear fraction*

The cells were harvested at the time desired, washed with cold PBS, scraped off the dish in 1 ml of 10 mM HEPES buffer, pH 7.4 and 0.25 mM sucrose. The cells were homogenized in a Bellco glass homogenizer using piston A (4 strokes). The supernatant after centrifugation at 800 r.p.m. for 5 min in the Sorval GLC 2B centrifuge was defined as the postnuclear fraction. This subcellular preparation was routinely used in this study. However, both the sedimentation pellet and the whole cell homogenate were also analyzed for enzyme activities.

### *Enzyme assays*

Palmitoyl-CoA hydrolase activity was assayed both radioactively and spectrophotometrically as described previously (Berge & Farstad, 1979; Berge & Døssland, 1979). The incubation medium contained 15 mM HEPES buffer, pH 7.4, 150 mM KCl, 2 mM EDTA, 0.01% (W/W) Triton X-100 and palmitoyl-CoA. When the spectrophotometric method was used, 0.3 mM DTNB was added in order to trap free CoASH. The protein content per assay was kept in the range from 5 to 50 $\mu$g. For further experimental details, see **Results**. The activities of cyanide-insensitive palmitoyl-CoA oxidase, catalase, urate oxidase and carnitine acetyltransferase were assayed according to earlier procedures (Berge & Bakke, 1981). Protein was determined using the Bio-Rad protein assay kit (Bio-Rad Lab., USA). Standard protein was bovine gamma globulin.

### *Reagents*

Basal Medium Eagle and heat-inactivated foetal calf serum were from Gibco, Scotland. Plastic petri dishes were either from Nunc, Denmark or from Costar, Mass., USA. Palmitoyl-CoA and cycloheximide were from Sigma Chemical Co. (St Louis, MO, USA).

[1-$^{14}$C] palmitoyl-CoA was purchased from New England Nuclear, Boston, MA, USA. TPA was obtained from PL/Biochemicals, USA. Tiadenol was a gift from Laboratorios Almirall, Barcelona, Spain. All other chemicals were of highest purity commercially available.

### Results

With a prepared postnuclear fraction of mouse embryo fibroblast C3H/10T1/2 C18 cells, the hydrolysis of palmitoyl-CoA was linear with the amount of protein up to ~50 $\mu$g (Figure 1). The rate of hydrolysis of palmitoyl-CoA by the corresponding cell fraction was constant up to ~6 min (data not shown). When the C18 cells were cultured in the presence of a non-toxic concentration of TPA (20 nM) for 9 days, the specific activity of the palmitoyl-CoA hydrolase activity (24.6 nmol min$^{-1}$ mg$^{-1}$ protein) was increased ~2.5 fold compared to control cells (9.7 nmol min$^{-1}$ mg$^{-1}$ protein) (Figure 1). Tiadenol administration, a potent hypolipidemic drug (Berge & Bakke, 1981) also enhanced this enzyme activity in the C18 cells and MCA16 cells (Berge & Lillehaug, 1985). When the C18 cells were cultured in the presence of 20 nM TPA for 24 h, the activity of the palmitoyl-CoA hydrolase activity declined ~30% (Figure 2). However, after this period there was a progressive increase in palmitoyl-CoA hydrolase activity up to 240 h. The palmitoyl-CoA hydrolase specific activity of untreated cells was constant during this growth period. For longer time of cell growth the hydrolase activity decreased

**Figure 1** Hydrolysis of palmitoyl-CoA in the post-nuclear fraction from C3H/10T1/2 C18 cells as a function of protein. The concentration of palmitoyl-CoA was 150 μM in a total volume of 0.25 ml. C3H/10T1/2 C18 cells were cultured for 9 days with 20 nM TPA (●); the control cultures received 0.5% acetone (○). The cells were treated during the logarithmic growth phase and the cultures reached confluence on day 9.

**Figure 2** Changes in the level of palmitoyl-CoA hydrolase (○) and catalase (●) in C18 cells treated with 20 nM TPA. The results are expressed as percent of control. The values are mean ± s.d. ($n=3$); □ represents the value of palmityol-CoA hydrolase activity obtained from TPA treated MCA16 cells. TPA treatment was started on the 4th day of culture. The cells were then in early log-phase growth (Berge & Lillehaug, 1985). The control values at time zero for palmitoyl-CoA hydrolase and catalase activities were 12 nmol min$^{-1}$ mg$^{-1}$ protein and 10 nmol min$^{-1}$ mg$^{-1}$ protein, respectively.

possibly due to a larger portion of the cells entering the $G_1/G_0$-phase (Berge & Lillehaug, 1985). The palmitoyl-CoA hydrolase activity was also enhanced in the chemically transformed C3H/10T1/2 MCA16 cells after TPA addition (Figure 2). Compared to control C18 cells the treatment of TPA decreased the catalase activity ~50% the first 120 h, but for longer incubation times (240 h) the specific activity of catalase, a peroxisome-associated enzyme, was enhanced ~1.8 fold.

When mouse embryo fibroblast C18 cells were grown in the presence of 20 nM TPA for up to 240 h, progressive increased activities of both cyanide-insensitive palmitoyl-CoA dehydrogenase and carnitine acetyltransferase (two specific enzymes for peroxisome proliferation) were obtained (Figure 3). The increase in the peroxisomal palmitoyl-CoA oxidation and carnitine acetyltransferase activity was evident 24 h after the addition of TPA. At 240 h, TPA treatments enhanced the peroxisomal-oxidation ~4-fold of control values. Compared to the catalase and palmitoyl-CoA hydrolase activities (Figure 2) no early inhibition of the activities of peroxisomal β-oxidation and carnitine acetyl-transferase was observed (Figure 3). Between 24 and 240 h, the level of palmitoyl-CoA oxidation in the control cultures decreased from ~0.4 nmol min$^{-1}$ mg$^{-1}$ protein to 0.15 nmol min$^{-1}$ mg$^{-1}$ protein. The activities of NADPH-cytochrome *c* reductase, a microsomal marker, and glutamate dehydro-genase, a mitochondrial marker, were not effected by TPA treatment up to 240 h (Figure 3). Similar results were obtained by TPA administration to MCA16 cells (data not shown).

**Figure 3** Changes in the levels of peroxisomal palmitoyl-CoA oxidation (△), carnitine acetyltransferase (▲), NADPH-cytochrome *c* reductase (□) and glutamate dehydrogenase (■) in C18 cells treated with 20 nM TPA. The control values (nmol min$^{-1}$ mg$^{-1}$ protein) at time zero for peroxisomal β-oxydation, carnitine acetyltransferase, NADPH-cytochrome C reductase and glutamate dehydrogenase were 0.3, 8.2, 1.8 and 20.4, respectively.

**Figure 4** Palmitoyl-CoA hydrolase activity and catalase activity in C18 cells and MCA16 cells at increasing concentrations of TPA. The cells were exposed to the drug for 10 days and fresh medium with drug was added 24 h before harvesting the cells. Palmitoyl-CoA hydrolase activity in the postnuclear fraction of C18 cells (●). Catalase activity in the post-nuclear fraction of C18 cells (■). Palmitoyl-CoA hydrolase activity in the whole homogenate of MCA16 cells (○). Catalase activity of the whole homogenate of MCA16 cells (□).

The results of dose-response studies with TPA are shown in Figure 4. TPA addition produced a dose-related increase in palmitoyl-CoA hydrolase activity over a concentration range of 5 to 20 nM in C18 and 5 to 50 nM in the MCA16 cells. As shown in Figure 4, TPA produced ~2.5 fold increase in the catalase activity in C18 cells at concentrations between 5 to 20 nM. TPA (5 nM) was sufficient to induce maximal catalase activity. Thus, MCA16 cells appear to be more sensitive than C18 cells in inducing peroxisome-associated enzymes. A dose-related increase in peroxisomal palmitoyl-CoA oxidation and carnitine acetyltransferase activity was also observed (data not shown). In the presence of 2.5 M cycloheximide which inhibits the protein synthesis by ~85% (Villa *et al.*, 1980), a substantial reduction in palmitoyl-CoA hydrolase activity and catalase activity was observed in cells treated both with TPA and tiadenol (Table I). This effect was obtained in C18 cells as well as in MCA16 cells. With the C18 cells, the stimulation of peroxisomal fatty acid oxidation obtained by TPA and tiadenol was reduced by cycloheximide. In the presence of cycloheximide, only a small reduction in carnitine acetyltransferase activity was observed after tiadenol and TPA treatment.

### Discussion

TPA is a strong tumour promoter and it has been used as a model promoter. Tumour promotion is a process in which the altered genotype of the initiated cell becomes expressed in such a way that oncogenic transformation occurs. Thus, although tumour promoters are considered to be epigenetic in action their effects are ultimately on the genome. Tumour promoters are not carcinogenic by themselves, but must work in sequence with an initiator.

**Table I** Effect of cycloheximide on the stimulation of peroxisomal enzyme activities by hypolipidemic drug treatment of cultured C3H/10T1/2 cells and chemically transformed cells, MCA16. The cycloheximide concentration was 1 $\mu g\,ml^{-1}$ and the duration of treatment was 24 h. The concentrations of TPA and triadenol were 18 nM and 10 μM, respectively

| | | Enzymic activities (nmol $min^{-1}$ $mg^{-1}$ protein) | | | | | |
|---|---|---|---|---|---|---|---|
| | | *Palmitoyl-CoA hydrolase* | | *Peroxisomal β-oxidation* | *Carnitine acetyl-transferase* | *Catalase* | |
| *Drug* | *Cyclo heximide 1 $\mu g\,ml^{-1}$* | *C18*[a] | *MCA16*[b] | *C18*[b] | *C18*[b] | *C18*[a] | *MCA16*[b] |
| Control | – | 15.1 | 20.1 | 0.25 | 9.6 | 9.2 | 8.5 |
| | + | 14.7 | 19.8 | 0.18 | 7.5 | 9.3 | 8.7 |
| TPA | – | 29.1 | 44.5 | 0.73 | 18.7 | 22.2 | 18.7 |
| | + | 16.4 | 18.2 | 0.22 | 17.0 | 13.1 | 8.2 |
| Triadenol | – | 22.1 | 48.0 | 0.55 | 12.9 | 20.7 | 14.9 |
| | + | 16.4 | 22.1 | 0.19 | 10.2 | 7.7 | 8.0 |

[a]The activity was measured in the nuclear fraction. [b]The activity was measured in the whole homogenate.

A number of systems have been proposed as models for *in vitro* promotion; of these mouse embryo fibroblast C3H/10T1/2 cells have been well characterized (Reznikoff *et al.*, 1973; Mondal & Heidelberger, 1976; Lillehaug & Djurhuss, 1982). Exposure of these cells at low density to carcinogenic agents such as polycyclic hydrocarbons, ultra-violet irradiation or X-rays at subcarcinogenic doses followed by sustained exposure to phorbol esters such as TPA results in augmented formation of transformed foci.

A marked stimulation of cyanide-insensitive palmitoyl-CoA oxidation, carnitine acetyltransferase and palmitoyl-CoA hydrolase activities with a moderate enhancement of the catalase activity, is characteristic of the preferential stimulation of peroxisomal enzymes associated with fatty acid metabolism produced *in vivo* by tiadenol, clofibrate, d(2-ethylhexyl)phthalate and other peroxisome proliferators (Berge & Bakke, 1981; Berge *et al.*, 1984; Bakke & Berge, 1982; Moody & Reddy, 1978). Recently and partly in this study we have shown that addition of tiadenol to cultures of C18 and MCA16 cells enhanced the corresponding enzyme activities (Berge & Lillehaug, 1985). Moreover, in the present study we have shown that addition of TPA to these mouse embryo fibroblasts enhanced the activities of peroxisomal-oxidation, carnitine acetyltransferase, palmitoyl-CoA hydrolase and catalase (incubation times between 120 and 240 h) while mitochondrial and microsomal marker enzymes showed little changes in activity. The first 120 h of cultivation with TPA decreased the catalase activity (Figure 2). This observation is in agreement with results from Solanski *et al.* (1981) reporting that TPA treatment for 16 h decreased the catalase activity in mouse epidermal cells. A similar time course was observed for the palmitoyl-CoA hydrolase activity. The initial inhibition of palmitoyl-CoA hydrolase and catalase activity may be related to the promoting activity of TPA while the stimulation observed during prolonged treatment may be explained, by the growth stimulating and the second stage tumour promoting properties of TPA. The inhibitory effect of cycloheximide on peroxisomal $\beta$-oxidation indicates that the increases produced in cell culture were largely the result of *de novo* protein synthesis. The low inhibitor effect of cycloheximide on the activity of carnitine acetyltransferase after TPA treatment may be explained by a stimulating effect on the enzyme itself rather than enhanced protein synthesis. Apart from being a peroxisome-associated enzyme, carnitine acetyltransferase is also localized to the mitochondria. The activity of glutamate dehydrogenase was not changed after TPA administration. Thus, these observations further complicate the interpretation of the effect of cycloheximide on carnitine acetyltransferase activity.

The two-stage carcinogenesis process has been studied in several *in vitro* cell culture assays. The culture system used in the present study is fibroblastic whereas well characterized two-stage systems are also epithelial. The end point analyzed in the present study seemed to be a late irreversible event in carcinogenesis since the foci are irreversibly growing and tumourigenic (Mondal & Heidelberger, 1976) in contrast to the papillomas produced in the well-characterized epidermal system *in vivo* which are reversibly growing (Burns *et al.*, 1978) and the cells from which are non-tumourigenic (Pera & Gorman, 1984). Moreover, TPA has not been demonstrated to influence any of the irreversible steps in epidermis *in vivo* (Verma & Boutwell, 1980). The first stage of promotion is reversible and melanomas are still promoted by TPA in species where papillomas are not (Sisskin & Barrett, 1981). Thus, these observations raise the question whether in general promoting agents produce similar effects in *in vivo* carcinogenesis and *in vitro* cell transformation.

Although we have not used electron microscopy for assessing these peroxisomal changes, the data suggest that the classical tumour promoter TPA has peroxisomal effects and may be classified as a peroxisome proliferator. Peroxisomes oxidize activated long-chain and medium-chain fatty acids and the removal of two carbons results in generation of one molecule of $H_2O_2$. Thus, $H_2O_2$ is generated as a by-product of the peroxisomal $\beta$-oxidation. The time course study demonstrated that the peroxisomal $\beta$-oxidation was enhanced greater and earlier than the catalase activity after TPA treatment e.g. after 180 h of cultivation the peroxisomal $\beta$-oxidation was increased $\sim$3-fold while the catalase activity was unaltered. Catalase is not so very effective in destroying $H_2O_2$ at low concentrations (Chance *et al.*, 1979). Thus, the $H_2O_2$ generated by peroxisomes can escape degradation by catalase. None of the tested carcinogenic peroxisome proliferators has been shown to be mutagenic in bacterial assays (Warren *et al.*, 1980). However, $H_2O_2$ and related oxygen free radicals are known to be mutagenic in repair-deficient strains of *Escherichia coli* (Demple *et al.*, 1983). Furthermore, $H_2O_2$ has also been shown to be carcinogenic in rats and mice. Furthermore, Fahl *et al.* (1984) have shown that DNA damage is related to increased $H_2O_2$ generation by hypolipidemic drug-induced liver peroxisomes. Recently, we have shown that hypolipidemic drugs such as clofibrate and niadenate, classified as strong peroxisome proliferators, show tumour promoting but no carcinogenic activity *in vitro* (Berge &

Lillehaug, 1985). As clofibrate induces hepatocellular tumours in both mice and rats when chronically administered in the diet (Reddy *et al.*, 1980), these observations raise the question whether mutagenic metabolites are generated by hypolipidemic drug-induced liver peroxisomes. Induction of hepatocellular tumours may be related to biologically active products of the proliferated peroxisome population rather than a direct drug effect. Therefore, the promoting activity of TPA, classified as a peroxisome proliferator, may be related to biologically active products of the proliferated peroxisome population e.g. $H_2O_2$.

In order to further assess the relevance of the data to tumour promotion and transformation, it will now be of interest to determine whether the number of foci in this culture system show a similar dose-dependence on TPA to the increased activity of peroxisomal $\beta$-oxidation.

The authors are grateful for the excellent assistance of Mrs A. Iden and H. Kaneström. This work was supported in part by the Norwegian Council for Science and Technocology (NTNF), the Norweigian Research Council for Science and the Humanities, the Norwegian Cancer Society and the Norweigian Society for Fighting Cancer.

### References

BACKER, J.M., BOERSIG, M.R. & WEINSTEIN, I.B. (1982). Inhibition of respiration by a phorbol ester tumor promoter in murine cultured cells. *Biochem. Biophys. Res. Commun.*, **105**, 855.

BAKKE, O.M. & BERGE, R.K. (1982). Lipid-metabolizing enzymes, CoASH and long-chain acyl-CoA in rat liver after treatment with tiadenol, nicotinic acid and niadenate. *Biochem. Pharmacol.*, **31**, 3930.

BERGE, R.K. & BAKKE, O.M. (1981). Changes in metabolizing enzymes of hepatic subcellular fractions from rats treated with tiadenol and clofibrate. *Biochem. Pharmacol.*, **30**, 2251.

BERGE, R.K. & FARSTAD, M. (1979). Dual localization of long-chain acyl-CoA hydrolase in rat liver: One in the microsomes and one in the mitochondrial matrix. *Eur. J. Biochem.*, **95**, 89.

BERGE, R.K. & DØSSLAND, B. (1979). Differences between microsomal and mitochondrial-matrix palmitoyl-coenzyme A hydrolase, and palmitoyl-L-carnitine hydrolase from rat liver. *Biochem. J.*, **181**, 119.

BERGE, R.K., HOSØY, L.H., AARSLAND, A., BAKKE, O.M. & FARSTAD, M. (1984). Enzymatic changes in rat liver associated with low and high doses of a peroxisome proliferator. *Toxicol. Appl. Pharmacol.*, **73**, 35.

BERGE, R.K., LILLEHAUG, J.R. (1985). Tiadenol mediated induction of peroxisomal enzymes in cultured C3H/10T1/2 C18 cells and in the chemically transformed C3H/10T1/2 MCA16 cells. *Int. J. Cancer* (in press).

BERGE, R.K., SKREDE, S. & FARSTAD, M. (1981). Effects of clofibrate on the intracellular localization of palmitoyl-CoA hydrolase and palmitoyl-L-carnitine hydrolase in rat liver. *FEBS Lett.*, **124**, 43.

BERGE, R.K., AARSLAND, A., BAKKE, O.M. & FARSTAD, M. (1983). Hepatic enzymes, CoASH and long-chain acyl-CoA in subcellular fractions as affected by drugs inducing peroxisomes and smooth endoplasmic reticulum. *Int. J. Biochem.*, **15**, 191.

BLUMBERG, P.M.(1981). *In vitro* studies on the mode of action of the phorbol esters, potent tumor promotors. *CRC. Crit. Rev. Tox.*, **8**, 199.

BURNS, F.J., VANDERLAAN, M., SNYDER, E. & ALBERT, R.E. (1978). Induction and progression kinetics of mouse skin papillomas. In: *Mechanisms of Tumor Promotion and Carcinogenesis*, Vol. 2, Slaga *et al.* (eds) p. 91. Raven Press, New York.

CHANCE, B., SIES, H. & BOVERIS, S. (1979). Hydroperoxide metabolism in mammalian organs. *Physiol. Rev.*, **59**, 527.

DEMPLE, B., HALBROOK, J. & LINN, S. (1983). Escherichia coli Xth mutants are hypersensitive to hydrogen peroxide. *J. Bacteriol.*, **153**, 1079.

FAHL, W.E., LALWANI, N.D., WATANABE, T., GOEL, S.K. & REDDY, J.K. (1984). DNA damage related to increased hydrogen peroxide generation by hypolipidemic drug-induced liver peroxisomes. *Proc. Natl Acad. Sci. (USA)*, **81**, 7827.

FITZGERALD, J.E., SANYER, J.L., SCHARDEIN, J.L., LAKE, R.S., McGUIRE, E.J. & DE LA IGLESIA, F.A. (1981). Carcinogen bioassay and mutagenicity studies with the hypolipidemic agent gemfibrozil. *J. Natl Cancer Inst.*, **67**, 1105.

GOLDSTEIN, I.M., ROOS, D., KAPLAN, H.B. & WEISSMAN, G. (1975). Complement and immunoglobins stimulate superoxide production by human leukocytes independently of phagocytosis. *J. Clin. Invest.*, **56**, 1155.

HICKS, R.M. (1983). Pathological and biochemical aspects of tumor promotion. *Carcinogenesis*, **4**, 1209.

INESTROSA, N.C., BRONFMAN, M. & LEIGHTON, F. (1979). Detection of peroxisomal fatty acyl-coenzyme A oxidase activity, *Biochem. J.*, **182**, 779.

KENSLER, T.W. & TRUSH, M.A. (1981). Inhibition of phorbol ester-stimulated chemiluminescence in human polymorphonuclear leukocytes by retinoic acid and 5,6-epoxyretionic acid. *Cancer Res.*, **41**, 216.

KLUWE, W.M., HASEMAN, J.K., FIELDING, D., DOUGLAS, J. & HUFF, J.E. (1982). The carcinogenicity of dietary di(2-ethylhexyl)phthalate (DEHP) in Fischer 344 rats and B6C3F micr. *J. Toxicol. Environ. Health*, **10**, 797.

KLUWE, W.M., HASEMAN, J.K. & HUFF, J.E. (1983). The carcinogenicity of di(2-ethylhexyl)phthalate (DEHP) in perspective. *J. Toxicol. Environ. Health*, **12**, 159.

LALWANI, N.D., REDDY, R.K., QURESHI, S.A. & REDDY, J.K. (1981). Development of hepatocellular carcinomas and increased peroxisomal fatty acid-oxidation in rats fed (4-chloro-6-(2,3-xylidino)-2-pyrimidinylthio)acetic acid (WY-14,643) in the semi-purified diet. *Carcinogenesis*, **2**, 645.

LEIGHTON, F., COLOMA, L. & KOENIG, C. (1975). Structure, composition, physical properties, and turnover of proliferated peroxisomes: A study of the tropic effects of SU-1 3437 on rat liver. *J. Cell. Biol.*, **67**, 281.

LILLEHAUG, J.R. & DJURHUS, R. (1982). Effect of diethylstilbestrol on the transformable mouse embryo fibroblasts C31+/10T1/2 CL8 cells. Tumor promotion, cell growth, DNA synthesis and ornithine decartoxylase. *Carcinogenesis*, **3**, 797.

LILLEHAUG, J., AARSÆTHER, N., BERGE, R.K. & MALE, R. (1985). Peroxisome proliferators show tumor promoting but no carcinogenetic activity *in vitro*. *Int. J. Cancer* (in press).

MONDAL, S. & HEIDELBERGER, C. (1976), Transformation of 10 T 1/2 Cl 8 mouse embyro fibroblasts by ultraviolet irradiation and a phorbol ester. *Nature*, **260**, 710.

MOODY, D.E. & REDDY, J.K. (1978). Hepatic peroxisome (microbody) proliferators in rats fed plasticizers and related compounds. *Toxicol. Appl. Pharmacol.*, **45**, 497.

PERA, M.F. & GORMAN, P.A. (1984). In vitro analysis of multistage epidermal carcinogenesis: development of indefinite renewal capacity and reduced growth factor requirements in colony forming keratinocytes precedes malignant transformation. *Carcinogenesis*, **5**, 671.

PRYME, I.F., LILLEHAUG, J.R., FJOSE, A. & KLEPPE, K. (1983). The nuclear associated endoplasmic reticulum is an early target for the action of the tumor promoter 12-O-tetradecanoylphorbol-13-acetate in 3H/10T1/2 fibroblasts. *FEBS. Lett.*, **152**, 17.

REDDY, J.K., AZARNOFF, D.L. & HIGNITE, C.E. (1980). Hypolipidemic hepatic peroxisome proliferators form a novel class of chemical carcinogen. *Nature*, **283**, 397.

REDDY, J.K., AZARNOFF, D.L., SVOBODA, D.J. & PRASAD, J.D. (1974). Nafenopin-induced hepatic microbody (peroxisome proliferation) and catalase synthesis in rats and mice: Absence of sex difference in response. *J. Cell. Biol.*, **61**, 344.

REDDY, J.K. & RAO, M.S. (1977). Malignant tumors in rats fed nafenopin, a hepatic peroxisome proliferator. *J. Natl Cancer Inst.*, **59**, 1645.

REDDY, J.K. & QURESHI, S.A. (1979). Tumorigenicity of the hypolipidemic peroxisome proliferator ethyl-*p*-chlorophenoxy isobutyrate (clofibrate) in rats. *Br. J. Cancer*, **40**, 476.

REDDY, J.K., LALWANI, N.D., REDDY, M.K. & QURESHI, S.A. (1982). Excessive accumulation of autofluorescent lipofuscin in the liver during hepatocarcinogenesis by methyl clotenapate and other hypolipidemic peroxisome proliferators. *Cancer Res.*, **42**, 259.

RESNIKOFF, C.A., BRANKOW, D.W. & HEIDELBERGER, C. (1973). Establishment and characterization of a cloned line of C3H mouse embryo cells sensitive to postconfluence inhibition of division. *Cancer Res.*, **33**, 3231.

SISSKIN, E.E. & BARRETT, J.C. (1981). Hyperplasia of Syrin Hamster epidermis induced by single but not multiple treatment with 12-O-tetradecarnoylphorbol-13-acetate. *Cancer Res.*, **41**, 346.

SOLANSKI, V., RANA, R.S. & SLAGA, T.J. (1981). Diminution of mouse epidermal superoxide dismutase and catalase activities by tumor promotor. *Carcinogenesis*, **2**, 1141.

VERMA, A.K. & BOUTWELL, R.K. (1980). Effects of dose and duration of treatment with the tumor-promoting agent 12-O-tetradecanoylphorbol-13-acetate on mouse skin. *Carcinogenesis*, **1**, 271.

VILLA, P., HOCKIN, L.J. & PAINE, A.J. (1980). The relationship between the ability of pyridine and substituted pyridines to maintain cytochrome P450 and inhibit protein synthesis in rat hepatocyte cultures. *Biochem. Pharmacol.*, **29**, 1773.

VON DANIKEN, A., LUTZ, W.K., JACK, R. & SCHLATTER, C. (1984). Investigation of the potential for binding of di-(2-ethylhexyl) phthalate (DEHP) and di-(2-ethylhexyl) adipate (DEHA) to liver DNA in vivo. *Toxicol. Applied, Pharmacol.*, **73**, 373.

WARREN, J.R., SIMMON, V.F. & REDDY, J.K. (1980). Properties of hypolipidemic peroxisome proliferators in the lymphocyte $^{3}$H-thymidine and salmonella mutagenesis assay. *Cancer Res.*, **40**, 36.